# Cytotoxicity of thymoquinone alone or in combination with cisplatin (CDDP) against oral squamous cell carcinoma *in vitro*

**DOI:** 10.1038/s41598-017-13357-5

**Published:** 2017-10-13

**Authors:** Omar M. Alaufi, Abdulwahab Noorwali, Fatheya Zahran, Ahmed M. Al-Abd, Safia Al-Attas

**Affiliations:** 1 Departement of Clinical Biochemistry, Faculty of Medicine, King Abdulaziz University, Jeddah, Saudi Arabia; 2 General Directorate of Medical Services, Ministry of Interior, Riyadh, Saudi Arabia; 3 Oral medicine and Periodontology Department, Faculty of Dentistry, Cairo University, Cairo, Egypt; 4 Pharmacology Department, Medical Division, National Research Centre, Cairo, Egypt; 5 Departement of Pharmacology and Toxicology, Faculty of Pharmacy, King Abdulaziz University, Jeddah, Saudi Arabia; 6 Nawah Scientific, Mokkatam, Cairo, Egypt; 7 Department of Oral Diagnostic Sciences, Faculty of Dentistry, King Abdulaziz University, Jeddah, Saudi Arabia

## Abstract

Cisplatin (CDDP) is potent anticancer agent used for several tumor types. Thymoquinone (TQ) is naturally occurring compound drawing great attention as anticancer and chemomodulator for chemotherapies. Herein, we studied the potential cytotoxicity of thymoquinone, CDDP and their combination against human oral squamous cell carcinoma cell in contrast to normal oral epithelial cells. CDDP similarly killed both head and neck squamous cell carcinoma cells (UMSCC-14C) and normal oral epithelial cells (OEC). TQ alone exerted considerable cytotoxicity against UMSCC-14C cells; while it induced weaker killing effect against normal oral epithelial cells (OEC). Equitoxic combination of TQ and CDDP showed additive to synergistic interaction against both UMSCC-14C and OEC cells. TQ alone increased apoptotic cell fraction in UMSCC-14C cells, as early as after 6 hours. In addition, prolonged exposure of UMSCC-14C to TQ alone resulted in 96.7 ± 1.6% total apoptosis which was increased after combination with CDDP to 99.3 ± 1.2% in UMSCC-14C cells. On the other hand, TQ induced marginal increase in the apoptosis in OEC and even decreased the apoptosis induced by CDDP alone. Finally, apoptosis induction results were confirmed by the change in the expression levels of p53, Bcl-2 and Caspase-9 proteins in both UMSCC-14c and OEC cells.

## Introduction

Oral cancer (subtype of head and neck cancer) is malignant neoplasm of either tongue, gingivae, lip, salivary glands, palate, floor of the mouth or buccal mucosa. Treatment options for head and neck cancers include surgery followed by adjuvant chemotherapy and/or radiotherapy^1,2^. Oral cancers are often detected at late stages, and patients with head and neck cancers usually had 58% chance of five-year survival rate. This low survival rate remains unfortunately unchanged over the last three decades. However, treating head and neck cancers in early stages might results in survival rate up to 80%^3–5^.

Nowadays researchers believed that alternative medicine has promising sources of new anticancer treatments^6^. Interestingly, the last few decades showed increased interest on the medicinal herbs or plants, because of their limited complications and fewer side effects compared to conventional chemotherapy^7^. Moreover, the World Health Organization urged and encouraged countries of the developing world to apply their traditional medicinal plant in their primary health care programs^8^. One of the most extensively studied medicinal plant and described as the “miracle herb of the century is Nigella sativa (NS)^9–11^. Nigella sativa from the family Ranunculaceae is an annual flowering plant also called black cumin, black seed, or Habbatul Barakah^10^. The crude oil and thymoquinone (TQ) extracted from its seeds have been folksy used for many centuries for the treatment of many human illnesses like cardiovascular complications, diabetes, asthma, kidney disease, oral diseases… etc., with medicinal effects that include anti-bacterial, anti-fungal, anti-viral, antihelminthic, anti-inflammatory, immunomodulatory and anti-cancer properties^11–13^.

Combination of cancer treatments possesses increased attention because it enhances the efficiency of the combined agents and decreases their toxicities by lowering the dose required for therapeutic benifit^14^. Cis-diamminedichloridoplatinum II (CDDP) is a chemotherapy drug under the name Cisplatin. CDDP is a member and the firstly released platinum-containing anticancer agents. CDDP and other platinum based chemotherapies such as, oxaliplatin and carboplatin, are widely used for different types of neoplasia^15^. It was a revolutionary anticancer drug, hereafter more than 150 years of CDDP glorification “drug of the 20th century,” clinical practice showed many serious side effects accompany its uses such as neurotoxicity, nephrotoxicity, ototoxicity, vomiting and nausea^16^.

Despite few studies for use of TQ in oral cancers, it showed promising anticancer properties^17–19^. The aim of the research is to investigate the effect of TQ alone or in combination with CDDP against human oral cancer cells (UMSCC-14) in comparison to their influence in normal epithelial cells (OEC) *in vitro*.

## Results

### Cell killing effect of TQ, CDDP and their combination against UMSCC-14C and OEC cell lines

**Figure 1:**
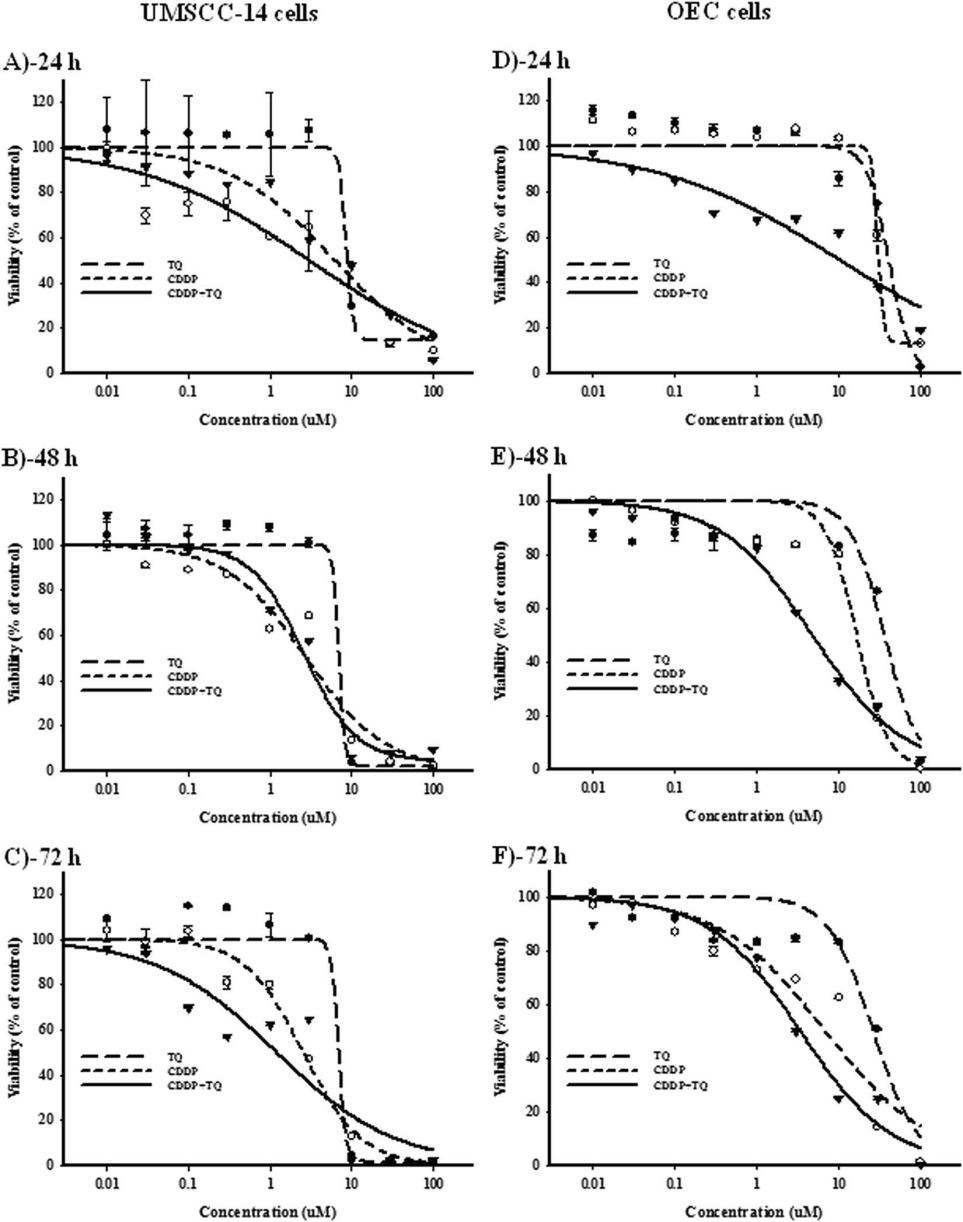
Dose response curves of CDDP (○), TQ (●) and their combination (▾) against UMSCC-14 (**A**–**C**) and OEC (**D**–**F**) cells after 24 h (**A** and **D**), 48 h (**B** and **E**) and 72 h (**C** and **F**) treatments.

The WST-1 assay was used to assess the viability of TQ, CDDP and their combination against UMSCC-14C (oral squamous carcinoma cells) and OEC (normal oral epithelial cells) cell lines over concentration range 0.01–100 μM. The viability parameters, IC_50_s and R-value were calculated using E_max_ model as described in the methods section. Treatment with TQ induced significant time and dose -dependent cytotoxic effects on UMSCC-14C cells (Fig. 1A–C). The IC_50_’s for TQ alone in UMSCC-14C cells for 24, 48 and 72 hours were 8.6 ± 0.4, 7.0 ± 2.3 and 7.0 ± 0.7 μM, respectively (Table 1). However, significantly less inhibitory effects were observed against the normal human oral epithelial cells (OEC) (Fig. 1D and E) with IC_50_’s concentrations equals’ 40.9 ± 7.4, 36.4 ± 1.9 and 26.8 ± 6.6 μM after 24, 48 and 72 hours of treatment, respectively (Table 1). On the other hand, CDDP alone was more potent cytotoxic than TQ against UMSCC-14C cells. CDDP alone showed significant inhibition of cell growth with IC_50_’s of 6.2 ± 0.4, 2.8 ± 1.1 and 2.6 ± 0.5 μM after treatment for 24, 48 and 72 hours, respectively (Fig. 1A–C, Table 1). Relatively, CDDP exerted lower cell killing effect against OEC cells with IC_50_’s of 30.5 ± 2.4, 16.8 ± 3.3 and 6.9 ± 0.7 μM after exposure to 24, 48 and 72 hours, respectively (Fig. 1D and E). Combination of TQ improved the cytotoxic effect of CDDP against UMSCC-14C cells and reduced its IC_50_’s to be 3.0 ± 0.6, 1.6 ± 0.7 and 1.2 ± 0.1 μM after exposure for 24, 48 and 72 hours, respectively (Fig. 1A–C, Table 1). Similarly, TQ increased the cell killing effect of CDDP against OEC cells with IC50’s of 10.1 ± 1.7, 4.8 ± 0.8 and 3.4 ± 0.9 μM after exposure for 24, 48 and 72 hours, respectively (Fig. 1A–C, Table 1). Combination analysis revealed additive killing effect between CDDP and TQ against UMSCC-14C cells with CI-values ranging from 0.8 to 0.97 at different exposure times. Unfortunately, the interaction between CDDP and TQ against OEC cells was synergistic (CI-values of 0.66 and 0.57) at early exposure time points (24 and 48 hours, respectively); and additive after 72 hour of exposure (Table 1).

**Table 1:** Temporal effects of TQ on the cytotoxicity parameters of CDDP in UMSCC-14 and OEC cell lines.

24 h exposure	UMSCC-14		OEC	
	IC_50_	R-value (%)	IC_50_	R-value (%)
CDDP	6.2 ± 0.4	14.6 ± 4.8	30.5 ± 2.4	13.1 ± 3.8
TQ	8.6 ± 0.4	2.6 ± 0.9	40.9 ± 7.4	2.7 ± 0.5
CDDP+TQ	3.0 ± 0.6	1.9 ± 0.3	10.1 ± 1.7	2.4 ± 0.6
**CI-value (24 h)**	**Additive/0.97**		**Synergism/0.66**	
**48 h exposure**	**IC**_**50**_	**R-value (%)**	**IC**_**50**_	**R-value (%)**
CDDP	2.8 ± 1.1	5.1 ± 0.7	16.8 ± 3.3	2.7 ± 0.8
TQ	7.0 ± 2.3	2.1 ± 0.4	36.4 ± 1.9	1.3 ± 0.2
CDDP+TQ	1.6 ± 0.7	3.9 ± 0.8	4.8 ± 0.8	4.5 ± 0.7
**CI-value (48 h)**	**Additive/0.8**		**Synergism/0.57**	
72 h exposure	**IC**_**50**_	**R-value (%)**	**IC**_**50**_	**R-value (%)**
CDDP	2.6 ± 0.5	9.6 ± 0.8	6.9 ± 0.7	2.5 ± 0.6
TQ	7.0 ± 0.7	1.6 ± 0.6	26.8 ± 6.6	3.3 ± 0.8
CDDP+TQ	1.2 ± 0.1	1.1 ± 0.1	3.4 ± 0.9	3.1 ± 0.7
**CI-value (72 h)**	**Additive/0.92**		**Additive/0.99**	

**Figure 2:**
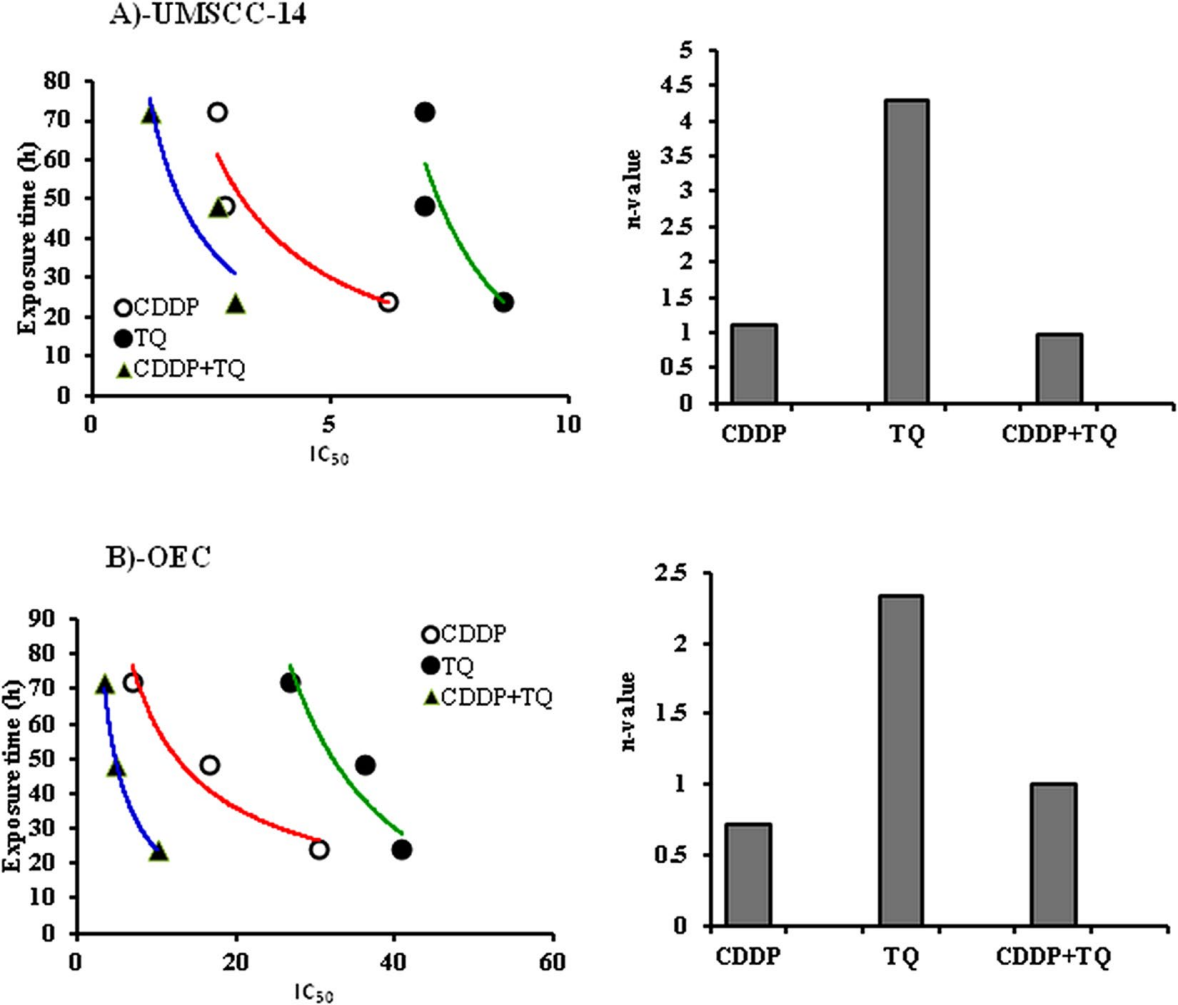
Temporal assessment for the cytotoxic profile of CDDP (○), TQ (●) and their combination (▴) against UMSCC-14 (**A**) and OEC (**B**) cells.

To determine the relative impact of cellular exposure time and concentration towards the killing effects of TQ, CDDP and their combination, C^n^T mathematical modeling was undertaken. As shown in Fig. 2A, n-value of 4.3 for TQ against UMSCC-14C cells indicates that TQ concentration is much more important for its killing effect compared to exposure time. In contrast, n-value of 1.1 for CDDP against UMSCC-14C cells indicates equivalent importance of both concentration and exposure time to the cell killing effect of CDDP. Combination of CDDP and TQ also has equivalent impact of both exposure time and concentration to UMSCC-14C cells with n-value of 0.97 (Fig. 2A). With respect to OEC cells, prolonged exposure to CDDP possesses higher influence on cell killing compared to concentration with n-value of 0.72. On the other hand, TQ concentration is the rate limiting step in its OEC cell killing effect rather than exposure time (n-value = 2.34). Interestingly, exposure time and concentration is equally impacting OEC cell killing effect for combined TQ and CDDP treatment (Fig. 2B).

#### Assessment of apoptosis

**Figure 3:**
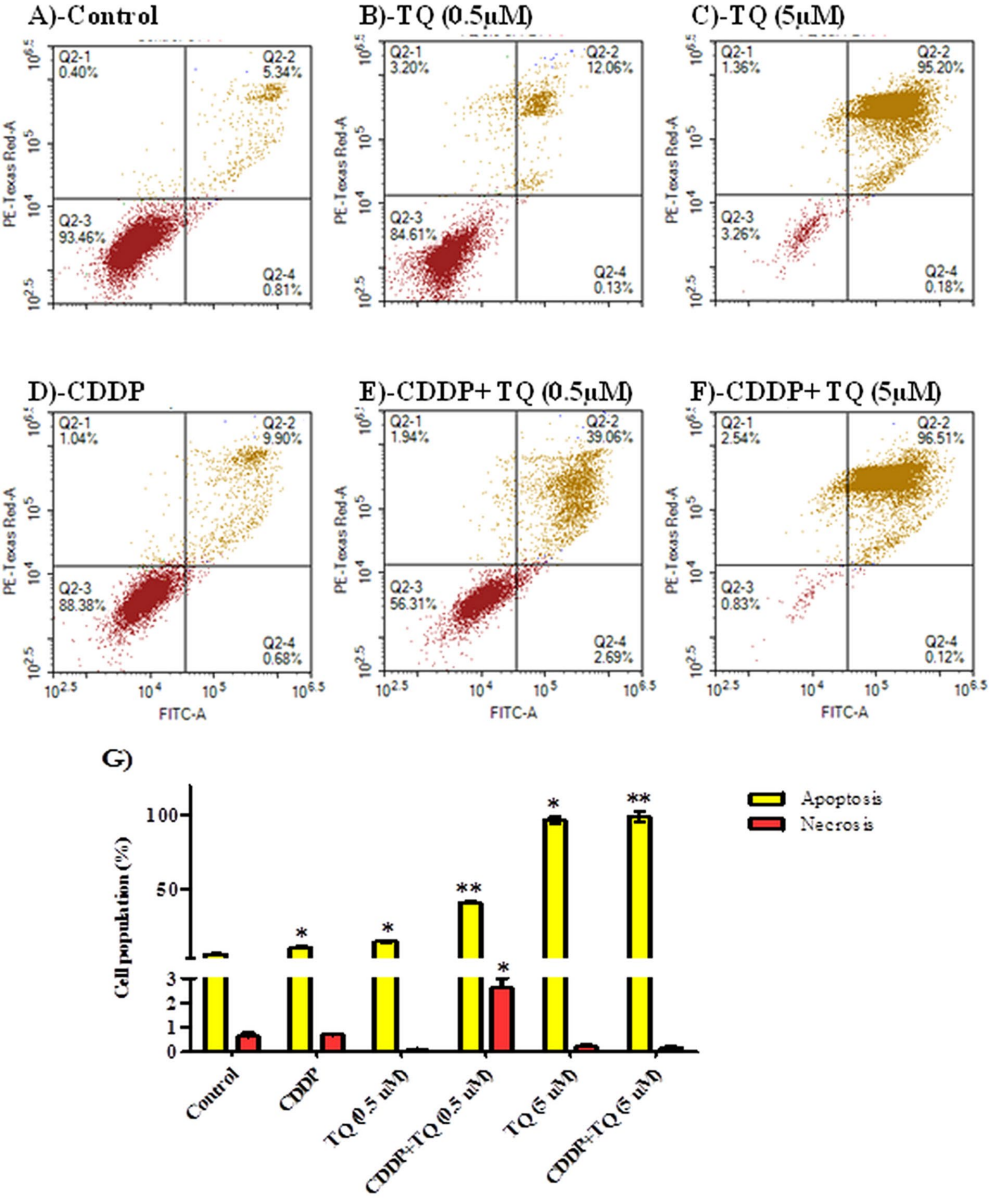
Mechanism of cell death induced by CDDP, TQ and their combination in UMSCC-14 cells. Cells were exposed to 0.5 µM TQ (**B**), 5 µM TQ (**C**), 5 µM CPDD (**D**), combination of 0.5 µM TQ+5 µM CPDD (**E**) and combination of 5 µM CPDD+5 µM TQ (**F**), for 24 h and compared to control cells (**A**). Cells were stained with annexin V-FITC/PI and different cell populations were plotted (**F**) as percentage of total events. Data is presented as mean ± SD; n = 3. ^*^Significantly different from control group. ^**^Significantly different from CDDP treatment.

**Figure 4:**
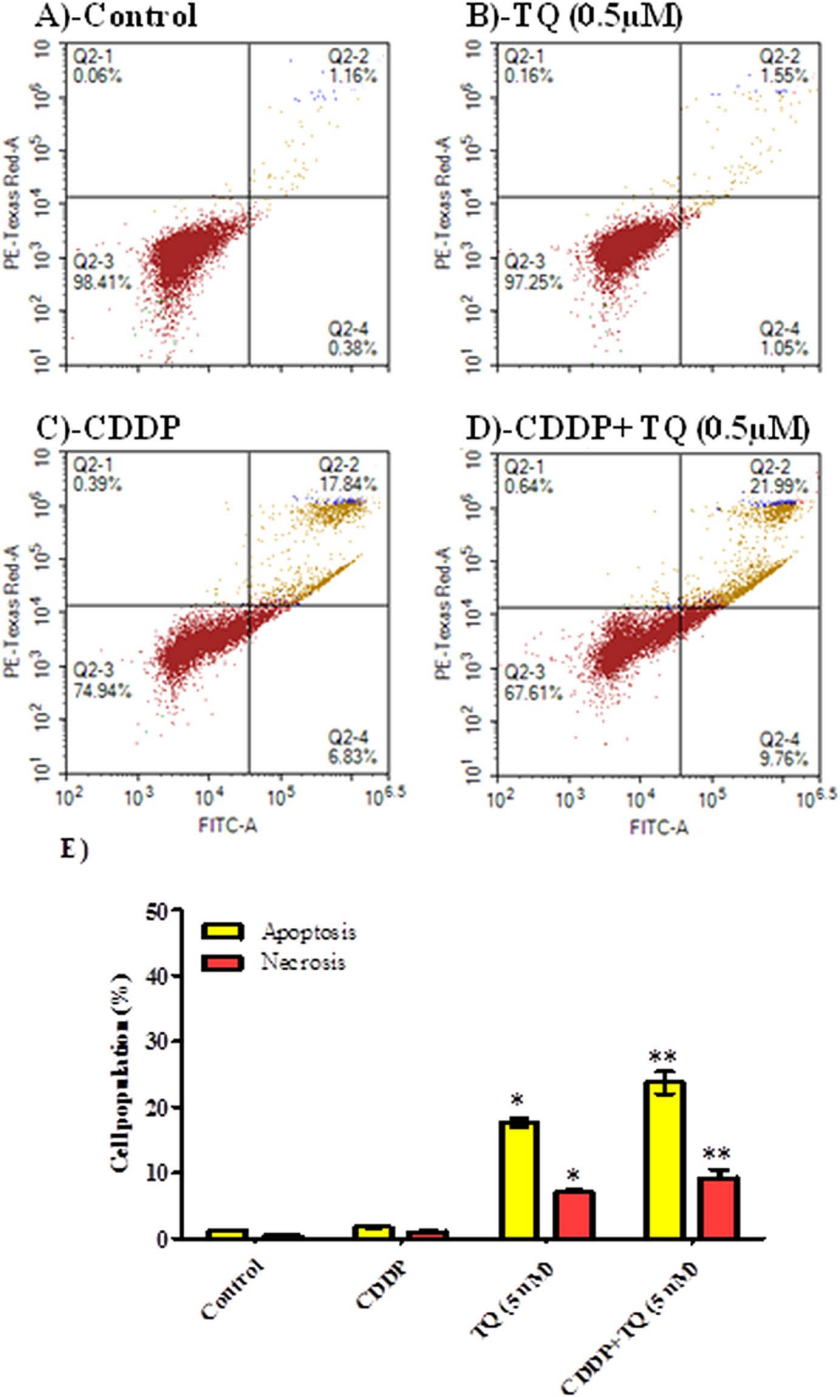
Mechanism of cell death induced by CDDP, TQ and their combination in UMSCC-14 cells. Cells were exposed to 5 µM TQ (**B**), 5 µM CPDD (**C**) and combination of 5 µM TQ+5 µM CPDD (**D**) for 6 h and compared to control cells (**A**). Cells were stained with annexin V-FITC/PI and different cell populations were plotted (**F**) as percentage of total events. Data is presented as mean ± SD; n = 3. ^*^Significantly different from control group. ^**^Significantly different from CDDP treatment.

To determine the exact mechanism of cell death (programmed or not programmed) induced by TQ, CDDP and their combination, cells were assayed by Annexin-V/FITC apoptosis detection assay after exposure to TQ (0.5 and 5 μM), CDDP (5 μM) and their combination for 6 and 24 hours treatment. TQ alone (0.5 and 5 μM) induced significant apoptosis in a dose dependent manner in UMSCC-14C cell line after 24 h exposure (14.4 ± 0.7% and 96.5 ± 2.6%, respectively) (Fig. 3A–C). CDDP (5 μM) induced apoptosis in UMSCC-14C cells after 24 hour (10.5 ± 0.7%); however less apoptosis induced by equimolar concentration of than TQ (Fig. 3A,C and D). Combination of CDDP (5 μM) with TQ (5 μM) induced apoptosis in 99.1 ± 3.4% of cells under treatment for 24 hours (Fig. 3F). Combination of CDDP (5 μM) with lower concentration of TQ (0.5 μM) induced apoptosis in 40.7 ± 0.8% of UMSCC-14C cells after 24 hours of treatment (Fig. 3E and G). Only combination of CDDP (5 μM) with of TQ (0.5 μM) induced significant necrosis in UMSCC-14C cells. In agreement with C^n^T modelling, TQ (0.5 and 5 μM) induced significant apoptosis (17.7 ± 0.7% and 23.7 ± 1.7%, respectively) and necrosis in UMSCC-14C cells as early as after 6 hours of exposure in a dose dependent manner. CDDP did not induce any significant apoptosis after this short exposure time (Fig. 4).

**Figure 5:**
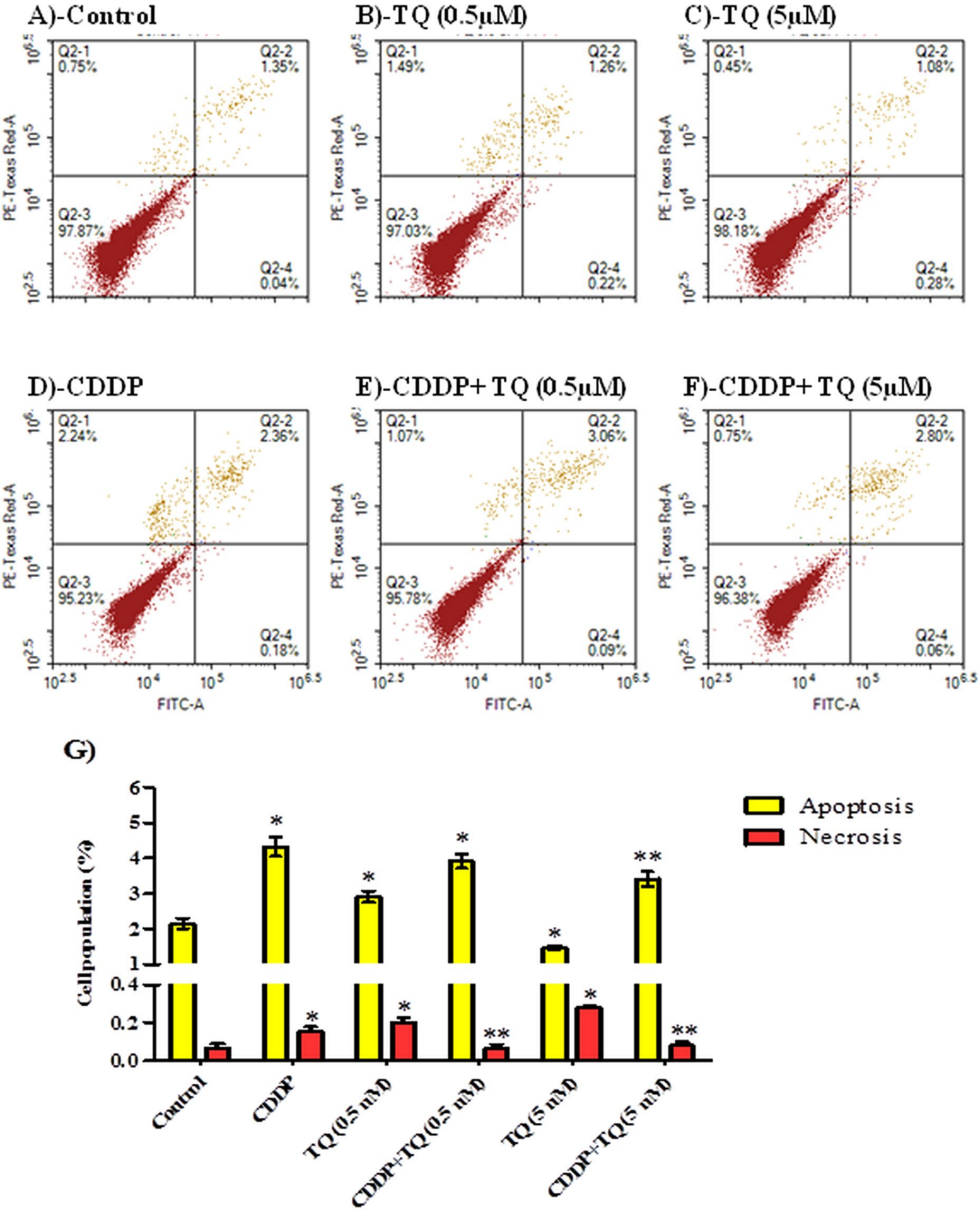
Mechanism of cell death induced by CDDP, TQ and their combination in OEC cells. Cells were exposed to 0.5 µM TQ (**B**), 5 µM TQ (C), 5 µM CPDD (**D**), combination of 0.5 µM TQ+5 µM CPDD (**E**) and combination of 5 µM CPDD+5 µM TQ (**F**), for 24 h and compared to control cells (**A**). Cells were stained with annexin V-FITC/PI and different cell populations were plotted (F) as percentage of total events. Data is presented as mean ± SD; n = 3. (^*^)Significantly different from control group. (^**^)Significantly different from CDDP treatment.

On the other hand, CDDP and TQ induced apoptosis and necrosis in OEC cell line as well (Fig. A–D). However, apoptosis induced by TQ within OEC cells was significantly less than UMSCC-14C cells. Besides, TQ (5 μM) significantly decreased CDDP induced apoptosis within OEC cells after 24 hour of exposure (Fig. 5D and F). In addition, TQ (0.5 and 5 μM) significantly protected from CDDP induced necrosis in OEC cells (Fig. 5D–G).

#### Western immunoblotting

**Figure 6:**
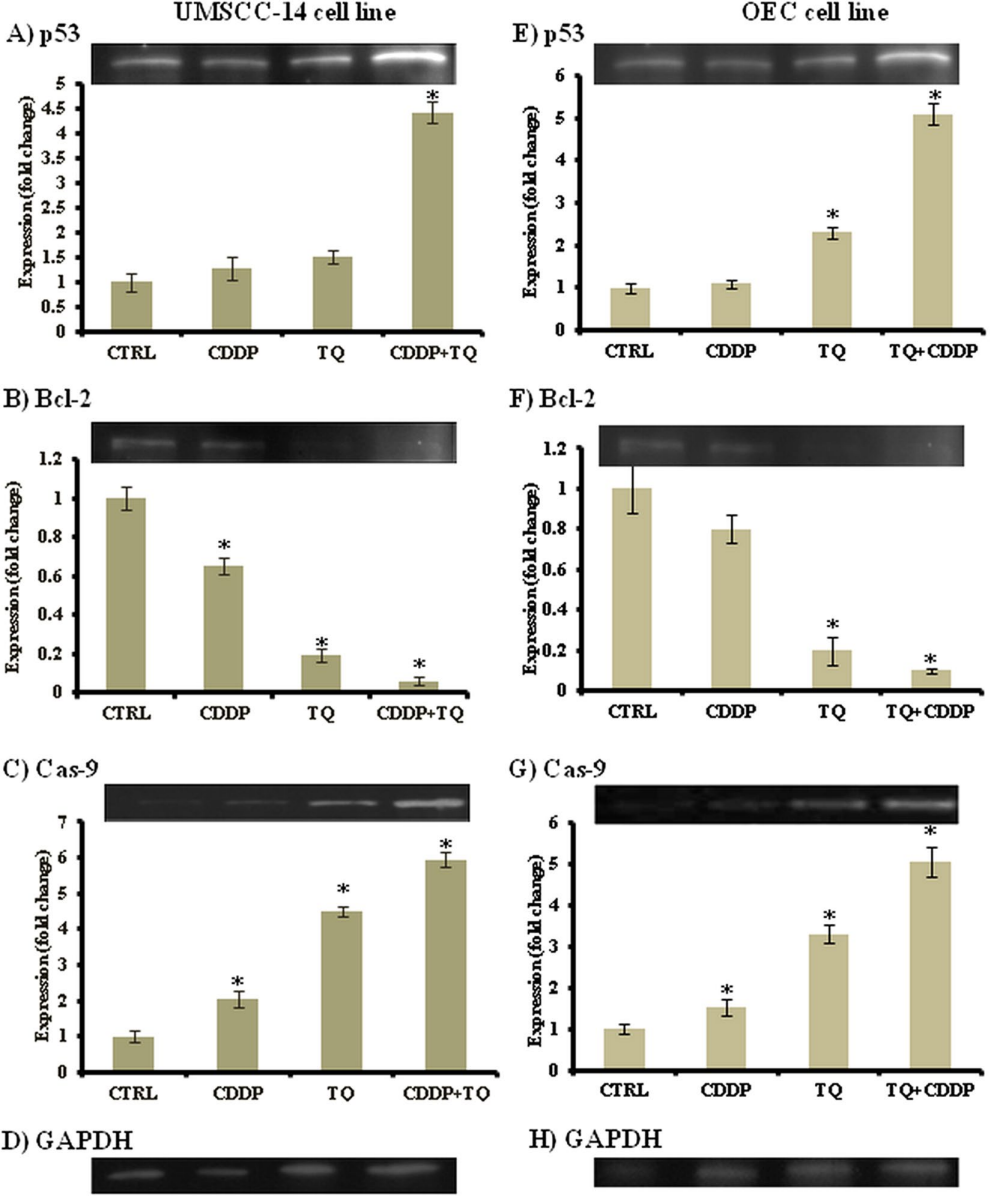
Effect of TQ, CDDP and their combination on expression level of p53 (**A** and **E**), Bcl-2 (**B** and **F**) and Caspase-9 (**C** and **G**) compared to GAPDH housekeeping gene (**D** and **H**) in UM-SCC-14C (**A**–**D**) and OEC (**E**–**H**) cell lines using western blot analysis. Data is presented as mean ± SD; n = 3. ^*^Significantly different from control group.

To further investigate the cytotoxic mechanism of action of TQ and its combination with CDDP against UMSCC-14C and OEC cells, Western immunoblotting was performed to assess the expression of the pro-apoptotic proteins (p53 and caspase-9) and the anti-apoptotic protein Bcl-2 after exposure to single and combination treatments. The results indicated that, treatment of UMSCC-14C and OEC cells with TQ and CDDP combination increases P53 expression compared to control by 4.4 and 5.1 folds, respectively. CDDP treatment alone increased P53 expression by only 1.5 folds compared to control cells of UMSCC-14C cells; while marginally increased its expression (1.1) in OEC cells (Fig. 6A and E). Thus combining TQ with CDDP caused around 3 and 5 folds increases in the latter’s effect on p53 expression within UMSCC-14C and OEC cells, respectively. Furthermore, TQ alone caused around 4.5 and 3 folds increases in caspase-9 expression compared to control in UMSCC-14C and OEC cells, respectively. Treatment with TQ and CDDP combination increased caspase-9 expression by 6 and 5 folds compared to control UMSCC-14C and OEC cells, respectively. Exposure to CDDP alone induced only 2 and 1.5 folds increased expression of caspase-9 in UMSCC-14C and OEC cells, respectively (Fig. 6B and F). Reciprocally, TQ alone decreased Bcl-2 expression by 5 folds in both UMSCC-14C and OEC cells compared to the untreated cells. CDDP showed a decrease by 35% and 20% in the expression of Bcl-2 compared to control UMSCC-14C and OEC cells, respectively. Combination of TQ with CDDP showed more decrease in the expression of Bcl-2 by 94% and 90% compared to control untreated UMSCC-14C and OEC cells, respectively (Fig. 6C and G).

## Discussion

Oral cancer although preventable, is considered a major health problem worldwide with variable extent. As it have increased morbidity and mortality as well as a low five year survival rate. Treatment options include surgery followed by adjuvant chemotherapy and radiotherapy^4,20^. Presently, platinum-based drugs (CDDP) such as cisplatin, remains one of the most commonly used chemotherapeutic agents available for the treatment of advanced oral cancers. While CDDP treatment often results in initial responses and disease stabilization, its long-term success is hindered by the development of drug resistance and dose-limiting toxicities^21^. In an attempt to find a safer, affordable and effective treatment option, we investigated here the use of thymoquinone (TQ) in oral cancer, one of the active components of *Nigella sativa* plant, traditionally used for various medicinal and nutritional purposes^12,22^. Also, we tested the hypothesis that combination of cisplatin and TQ may result in a more noticeable anticancer effect in oral cancer when compared to either agent alone using UMSCC-14C oral cancer cells in an *in vitro* study. This is the first study of the effect of TQ with cisplatin in oral cancer to the best of our knowledge.

The results revealed a dose and time dependent cytotoxic effects and decrease of the viability of UMSCC-14C oral cancer cells in response to TQ treatment. Moreover, TQ showed negligible cytotoxic effects on human normal oral epithelial cell (OEC) in low concentrations. TQ alone showed significant antiproliferative/cytotoxic effects but it was not as potent as CDDP. Cell killing effect of TQ was more concentration-dependent while cell killing effect of CDDP was more time-dependent. However, the combined cytotoxic effect of TQ and CDDP was both concentration- and time-dependent. Interestingly, TQ enhanced the cytotoxic effects of CDDP against both normal and cancer cells. However there was noticeable safety margin (about 3 folds) between the combinations IC_50_’s in both cell lines. In other words the killing effect of CDDP and TQ was 3 folds more potent in UMSCC-14 cells than OEC cells. It is disappointing to find out that the safety margin of CDDP killing effect was ranging from 2.8–6 folds between both tumor and normal cell lines (UMSCC-14 and OEC, respectively).

The literatures reported many *in vivo* and *in vitro* studies that showed significant TQ anticancer properties in different types of tumor cells and malignancies^23,24^. It was found to be safe and effective against many cancers, such as lung, kidney, liver, prostate, blood, cervical and skin cancers^23^. More importantly, authors consider it one of the ideal cancer therapeutic agents due to exerting anti-cancer effects with little cytotoxic effects to normal cells^13,25^. In agreement with other studies, the current work revealed that TQ could be considered relatively safe on non-tumor cells with reciprocal considerable activity against tumor cells^19,26,27^.

Studies that investigated TQ effects on oral squamous cell carcinoma were scanty^17–19^. Earlier in 2010, Rajkamal *et al*. investigated the chemopreventive effect of TQ in oral cancers *in vivo*. They reported that, oral administration of TQ at a dose of 30 mg/kg body weight reduces the tumor formation in the cheek pouches of DMBA painted hamsters. On the other hand, Abdelfadil and colleagues studied the TQ on unique cell lines developed by their lab representing chemically induced oral cancer cells (T28). They found that, the anti-cancer activity of TQ may be attributed to the downregulation of p38β MAPK. Later on in 2014, Chu and his colleagues reported that, TQ elicited a strong cytotoxic effect on a highly malignant HNSCC cell line SASVO3. Also, similar to us they indicated that, the TQ cytotoxic effect were mainly concentration-dependent. We can say that, the results of current research further prove the promising effect of TQ in oral cancer treatment. Although, it was the first time to be investigated in oral cancer, the ability of TQ to cause synergistic cytotoxicity in combination with chemotherapeutic agents such as 5-fluorouracil, doxorubicin and CDDP were reported in other cell lines^28–30^. In addition, it was reported that, TQ ameliorates the drugs complications, as it improves the CDDP-induced nephrotoxicity and doxorubicin-induced cardiotoxicity in animal models, most probably by its anti-oxidant activity^31^.

The molecular mechanisms behind the TQ anticancer effect are still not clearly understood^24^, however, some studies proposed that TQ has anticancer effect as it have antioxidant role and it improves body’s defence system, induces apoptosis and controls Akt pathway^23^. Herein we investigated the potential apoptotic effect of TQ against UMSCC-14C cells using Annexin-V staining. Then we screened the expression of the pro-apoptotic proteins (p53 and caspase-9) and the anti-apoptotic protein Bcl-2 after exposure to TQ, CDDP and their combination. Our observation showed that both TQ and CDDP induced apoptosis in UMSCC-14C cells. However, TQ alone or in combination with CDDP showed marked elevated rates of apoptosis compared to control cells or CDDP-treated cells alone. Interestingly, TQ induced apoptosis in UMSCC-14C cells as early as after only 6 hours of exposure. This finding fortifies and confirms our previous C^n^xT temporal analysis which showed higher importance of TQ concentration than exposure time in terms of cell killing effect. Moreover, TQ counteracted the apoptotic and necrotic activities of CDDP against OEC cells. Although, the current findings demonstrated a cytotoxic synergistic effect between TQ and CDDP in normal epithelial cells, it might be only at higher doses such as reported IC_50_’s and higher. This also can be explained by the greater impact of TQ concentration on its killing effect to both normal and cancer cells. In general, further research for TQ influence to molecular and submolecular signalling pathways of different normal cell lines is highly recommended. In particular, it is recommended to investigate the molecular mechanisms and signalling pathways involved in TQ induced cyto-protective effects to normal cells.

Apoptosis or programmed cell death is considered an ideal target in cancer therapy^19,32^. The p53 is a key factor in initiating apoptosis in response to DNA damage, which is critical for cancer survival^17^, while Bcl-2 family proteins are important in apoptosis regulation by acting as either promoters (e.g., Bax) or inhibitors (e.g., Bcl-2) of programmed cell death^19^. The current work, we showed a significant upregulation of p53, as well as apoptosis executive protein, caspase-9. On the other hand, there was decrease in the antiapoptotic Bcl-2 protien expression. These results could explain the potential mechanism of apoptosis induction in response to TQ treatment. Yet, TQ improved all CDDP induced apoptotic effects against both normal (OEC) and cancer (UMSCC-14C) cells.

Overall, our results provided further evidence of apoptotic activities of TQ in cancer cells, which agreed with several reports in treatment of different cancer cell lines such as myeloblastic leukemia HL-60 cells^33^, colon cancer cell line HCT116^34^ and pancreatic cancer cell^35^. Moreover, the current work supports previous findings on the mechanism of action of the TQ in oral cancer cells. It was reported that, TQ induced apoptotic cell death in SASVO3 cells by increasing Bax expression and caspase-9 activation^17^. Additionally, TQ increased the expression and activation of p53 within T28 oral cancer cells^19^.

In conclusion, TQ and CDDP, alone or in combination, inhibit cell viability and induce apoptosis in oral squamous cell carcinoma cells (UMSCC-14C). TQ induces apoptotic cell death in oral cancer cell lines by upregulating the expression of apoptotic genes (p53 and caspase 9) and down-regulating the expression of anti-apoptotic genes (e.g., Bcl 2). In addition, the cytotoxic effect of either TQ or CDDP against normal oral epithelial cells (OEC) is much milder than against cancer cells of the same origin. Combination TQ and CDDP is promising as an anti-head and neck cancer strategy; however, it must be taken with care due to higher cytotoxic risk against normal oral epithelial cells. Further molecular researches for such combination are highly urged before recommending it to clinical application.

## Materials and Methods

### Chemicals and drugs

Thymoquinone (TQ) and Cisplatin (CDDP) were purchased from Sigma Chemical Co. (St. Louis, MO, USA). RIPA lysis buffer kit was purchased from (Santa Cruz Biotechnology, Santa Cruz, CA, USA). Media, fetal bovine serum and other cell culture materials were purchased from Gibco™, Thermo Fisher scientific (Waltham, MA, USA). ECL detection system was purchased from Bio-Rad, Hercules, CA, USA. Pierce™ BCA protein assay kit was purchased from Thermo Fisher scientific (Waltham, MA, USA). Annexin-V/FITC apoptosis detection kit purchased from (BD Biosciences, San Jose, CA, USA) and WST-1 cell viability assay kit was purchased from (Abcam, Cambridge, UK). Other reagents were of the highest analytical grade.

### Cell culture

University of Michigan human mouth squamous cell carcinoma cell lines (UMSCC-14C was purchased from Cell Lines Service CLS, Eppelheim, Germany. Human normal oral epithelial cell line (OEC) was purchased from Applied Biological Materials Inc. Crestwood Place, Canada.OEC cells were maintained as monolayer cultures in DMEM with 10% fetal bovine serum supplementation, penicillin-streptomycin, L-glutamine, non-essential amino acids. UMSCC-14C cells were maintained as monolayer cultures in (1:1 mixture) DMEM: Ham’s F12 medium supplemented with L-glutamine and 5% fetal bovine serum. All cell lines were maintained in humidified incubator with 5% CO2 at 37 °C.

### Cell viability assay

Cellular viability and cell growth of UMSCC-14C and OEC cells were tested after treatment with TQ, CDDP, and their combination using the WST-1 cell viability assay kit according to the manufacturer’s instructions. WSTs are highly water-soluble tetrazolium salts. It produces water-soluble formazans and is suitable for cytotoxicity assays and cell proliferation. Briefly, cells under investigation were trypsinized, and proper dilution in the compatible media was made. Aliquots of 100 μl cell suspension containing 1000–2000 cells were seeded into the flat-bottom 96-well plate. Other aliquots of 100 μl media containing the drug concentration range (0.01 to 100 µM) under investigation were added to treated lanes, and blank media to the +ve, and −ve control lanes. Negative control is the well contains only media while positive control is the well contains cells but not treated. Plates were incubated in humidified 37 °C, 5% CO2 chamber for 24, 48 and 72 hrs. On the day of analysis, 10 µl WST-1 reagents was added to each well, and then the plates were incubated at 37 °C for 30 minutes to 4 hours. Then the plates were shaken for 1 min. Plates were read using Micro-Plate reader at 420–480 nm and the reference wavelength was ~650 nm. Relative WST-1 absorbance was measured using Syva Autotrak EIA Autoreader (Bio-Tek Instruments, Inc., Winooski, VT, USA). There were six wells for each condition and experiment was repeated triple to validate the results.

### Data analysis

The dose response curve of the compounds was analyzed and the IC_50_ concentration of the drugs determination was fitted to E_max_ model according to the following equation:

1 $$ \% \,{\rm{Cell}}\,{\rm{viability}}=(100-{\rm{R}})\times (1-\frac{{[{\rm{D}}]}^{{\rm{m}}}}{{{\rm{K}}}_{{\rm{d}}}^{{\rm{m}}}-{[{\rm{D}}]}^{{\rm{m}}}})+{\rm{R}}$$

where R is the resistance fraction, [D] is the concentration of drug, K_d_ is the concentration of the drug that makes the reduction of the maximum inhibition rate by 50%, and m is the “Hill-type coefficient”. IC_50_ is defined as the concentration of the drug required to reduce color intensity by 50% of that of the control^36^.

On the other hand, for drugs combination assessment the combination index (CI) was calculated as described by Chou and Talalay 1984^37^ using the following formula:

2 $$CI=\frac{I{C}_{50}\,of\,drug(x)\,combination}{I{C}_{50}\,of\,drug(x)\,alone}+\frac{I{C}_{50}\,of\,drug(y)\,combination}{I{C}_{50}\,of\,drug(y)\,alone}$$

The nature of drug interaction is considered synergism if CI < 0.8 or antagonism if CI > 1.2; and additive if CI ranges from 0.8–1.2.

The relationship between concentration, exposure time, and cytotoxic effect was analyzed using C^n^T model as previously described^38^.

K = C^n^ × T, Where, C is the drug concentration, T is the exposure time, n is the drug concentration coefficient, and K is the drug effect. Regression analysis was used for model fittings. This model was used to determine the differential influence of drug concentration and exposure time components on the cytotoxic effect of single or combined treatment. When n > 1, the impact of drug concentration is more influential than time of exposure, and vice versa.

### Apoptosis assessment using annexin V-FITC/PI staining coupled with flowcytometry

To assess the effect of CDDP, TQ and their combination on programmed cell death, apoptosis and necrosis cell populations were determined using Annexin V-FITC apoptosis detection kit (Abcam Inc., Cambridge Science Park, Cambridge, UK). Briefly, cells were treated with 0.5 µM and 5 µM TQ and/or 5 µM CDDP for 6 and 24 h. cells were collected by trypsinization, washed twice with ice-cold PBS, and re-suspended in 0.5 ml of annexin V-FITC/PI solution for 30 min in dark according to manufacturer protocol. After staining at room temperature, cells were injected through ACEA Novocyte™ flowcytometer (ACEA Biosciences Inc., San Diego, CA, USA) and analyzed for FITC and PI fluorescent signals using FL1 and FL2 signal detector, respectively (λ_ex/em_ 488/530 nm for FITC and λ_ex/em_ 535/617 nm for PI). For each sample, 12,000 events were acquired and positive FITC and/or PI cells were quantified by quadrant analysis and calculated using ACEA NovoExpress™ software (ACEA Biosciences Inc., San Diego, CA, USA).

### Assessment of protein expression using Western Blot Analysis

Cellular expression of key pro-apoptotic proteins (p53 and caspase-9) and anti-apoptotic protein Bcl-2 were assayed by Western immunoblotting after exposure to CDDP, TQ and their combination. Whole-cell lysates were prepared from treated UMSCC-14C and OEC cell lines after exposure to the IC_50_ values of CDDP, TQ and their combination for 24 hours then incubated with RIPA lysis buffer kit. Protein concentrations were quantified using Pierce™ BCA Protein Assay Kit and all protein lysates were adjusted to equal concentration. Electrophoresis on 12.5% SDS-polyacrylamide gel separated the whole-cell proteins (30 µg) and transferred to a polyvinylidene difluoride membrane (PVDF). Then membranes were sequentially probed with antibodies against the following proteins (Bcl-2, caspase-9, and p53). GAPDH was used as housekeeping internal standard protein (loading control). Membranes were washed; and blots were incubated with horseradish peroxidase-conjugate with appropriate secondary antibodies for an hour at 20 °C. The signals of each blot were developed and visualized using ECL detection Kit. Bands’ intensities were normalized by their corresponding GAPDH bands’ intensities. Bands with normalized relative intensities were quantified using ImageJ v1.43 analysis software (NIH, USA).

### Statistical analysis

Statistical analysis was presented as the mean ± standard deviation (SD) of at least three experiments. The maximum agonist response (E_max_) was calculated from concentration – response curve by nonlinear regression analysis of individual curves and IC_50_’s calculation were carried out using a computer-based fitting program (Sigma Plot version 16.0). Paired 2-sided test, one-way, and two-way analysis of variance (ANOVA) were used for data analysis. A *P* value of <0.05 was considered statistically significant.

## Electronic supplementary material

Supplementary File

## References

[1] Lingen MW, Kalmar JR, Karrison T, Speight PM (2008). Critical evaluation of diagnostic aids for the detection of oral cancer. Oral oncology.

[2] Rivera C (2015). Essentials of oral cancer. International journal of clinical and experimental pathology.

[3] Lag, R. *et al*. Seer cancer statistics review. *Bethesda, National Cancer Institute*, 1975–2003 (1975).

[4] Siegel RL, Miller KD, Jemal A (2016). Cancer statistics, 2016. CA: a cancer journal for clinicians.

[5] Torre LA (2015). Global cancer statistics, 2012. CA Cancer J Clin.

[6] Cragg GM, Grothaus PG, Newman DJ (2009). Impact of natural products on developing new anti-cancer agents. Chemical reviews.

[7] Dev S (2010). Impact of natural products in modern drug development. Indian J Exp Biol.

[8] WHO, W. H. O. WHO traditional medicine strategy 2002–2005. (2002).

[9] Ahmad I, Tripathi J, Sharma M, Karchulli M, Umer L (2014). Nigell a sativa-a medicinal herb with immense therapeutic potentia l (a systematic review). International Journal of Biological & Pharmaceutical Research.

[10] Ahmad A (2013). A review on therapeutic potential of Nigella sativa: A miracle herb. Asian Pacific journal of tropical biomedicine.

[11] AlAttas SA, Fat’heya MZ, Turkistany SA (2016). Nigella sativa and its active constituent thymoquinone in oral health. Saudi medical journal.

[12] Rooney S, Ryan M (2005). Modes of action of alpha-hederin and thymoquinone, active constituents of Nigella sativa, against HEp-2 cancer cells. Anticancer research.

[13] Darakhshan S, Pour AB, Colagar AH, Sisakhtnezhad S (2015). Thymoquinone and its therapeutic potentials. Pharmacological Research.

[14] Aggarwal BB (2006). From traditional Ayurvedic medicine to modern medicine: identification of therapeutic targets for suppression of inflammation and cancer. Expert opinion on therapeutic targets.

[15] Apps MG, Choi EH, Wheate NJ (2015). The state-of-play and future of platinum drugs. Endocrine-related cancer.

[16] Giaccone G (2000). Clinical perspectives on platinum resistance. Drugs.

[17] Chu S-C, Hsieh Y-S, Yu C-C, Lai Y-Y, Chen P-N (2014). Thymoquinone induces cell death in human squamous carcinoma cells via caspase activation-dependent apoptosis and LC3-II activation-dependent autophagy. PloS one.

[18] Rajkamal G, Suresh K, Sugunadevi G, Vijayaanand M, Rajalingam K (2010). Evaluation of chemopreventive effects of Thymoquinone on cell surface glycoconjugates and cytokeratin expression during DMBA induced hamster buccal pouch carcinogenesis. BMB reports.

[19] Abdelfadil E (2013). Thymoquinone induces apoptosis in oral cancer cells through p38β inhibition. The American journal of Chinese medicine.

[20] Day TA (2003). Oral cancer treatment. Current treatment options in oncology.

[21] In LL (2012). 1′-Acetoxychavicol acetate inhibits growth of human oral carcinoma xenograft in mice and potentiates cisplatin effect via proinflammatory microenvironment alterations. BMC complementary and alternative medicine.

[22] Shrivastava R, Agrawal R, Parveen Z (2011). A review on therapeutic applications of Nigella sativa. J Chem Chem Sci.

[23] Khan, A., Chen, H., Tania, M. & Zhang, D. Anticancer activities of Nigella sativa (black cumin). *African Journal of Traditional, Complementary and Alternative Medicines* **8** (2011). 10.4314/ajtcam.v8i5S.10 PMC3252704 22754079

[24] Rahmani, A. H., Alzohairy, M. A., Khan, M. A. & Aly, S. M. Therapeutic implications of black seed and its constituent thymoquinone in the prevention of cancer through inactivation and activation of molecular pathways. *Evidence-Based Complementary and Alternative Medicine* **2014** (2014). 10.1155/2014/724658 PMC4052177 24959190

[25] Shoieb AM, Elgayyar M, Dudrick PS, Bell JL, Tithof PK (2003). *In vitro* inhibition of growth and induction of apoptosis in cancer cell lines by thymoquinone. International journal of oncology.

[26] Kanter M, Akpolat M, Aktas C (2009). Protective effects of the volatile oil of Nigella sativa seeds on β-cell damage in streptozotocin-induced diabetic rats: a light and electron microscopic study. Journal of molecular histology.

[27] Al-Ali A, Alkhawajah AA, Randhawa MA, Shaikh NA (2008). Oral and intraperitoneal LD50 of thymoquinone, an active principle of Nigella sativa, in mice and rats. J Ayub Med Coll Abbottabad.

[28] Lei X (2012). Thymoquinone inhibits growth and augments 5-fluorouracil-induced apoptosis in gastric cancer cells both *in vitro* and *in vivo*. Biochemical and biophysical research communications.

[29] Effenberger-Neidnicht K, Schobert R (2011). Combinatorial effects of thymoquinone on the anti-cancer activity of doxorubicin. Cancer chemotherapy and pharmacology.

[30] Jafri SH (2010). *Thymoquinone and cisplatin as a therapeutic combinati*on in lung cancer: *In vitro* and *in vivo*. Journal of Experimental & Clinical Cancer Research.

[31] Badary OA, Taha RA, Gamal El-Din AM, Abdel-Wahab MH (2003). Thymoquinone is a potent superoxide anion scavenger. Drug and chemical toxicology.

[32] Hwang J-M (2012). KHC-4 anti-cancer effects on human PC3 prostate cancer cell line. The American journal of Chinese medicine.

[33] El‐Mahdy MA, Zhu Q, Wang QE, Wani G, Wani AA (2005). Thymoquinone induces apoptosis through activation of caspase‐8 and mitochondrial events in p53‐null myeloblastic leukemia HL‐60 cells. International journal of cancer.

[34] Halagali-Muhtasib M-A (2004). Thymoquinone extracted from black seed triggers apoptotic cell death in human colorectal cancer cells via a p53-dependent mechanism. International journal of oncology.

[35] Torres MP (2010). Effects of thymoquinone in the expression of mucin 4 in pancreatic cancer cells: implications for the development of novel cancer therapies. Molecular cancer therapeutics.

[36] Mahmoud AM, Al-Abd AM, Lightfoot DA, El-Shemy HA (2012). Anti-cancer characteristics of mevinolin against three different solid tumor cell lines was not solely p53-dependent. Journal of enzyme inhibition and medicinal chemistry.

[37] Chou TC, Talalay P (1984). Quantitative analysis of dose-effect relationships: the combined effects of multiple drugs or enzyme inhibitors. Advances in enzyme regulation.

[38] Levasseur LM, Slocum HK, Rustum YM, Greco WR (1998). Modeling of the time-dependency of *in vitro* drug cytotoxicity and resistance. Cancer Res.

