# Supplementary material for: Cytotoxicity of thymoquinone alone or in combination with cisplatin (CDDP) against oral squamous cell carcinoma in vitro
Source: Sci Rep. 2017 Oct 13;7:13131. doi: 10.1038/s41598-017-13357-5 (PMC5640598; doi:10.1038/s41598-017-13357-5)

Cytotoxicity of thymoquinone alone or in combination with cisplatin (CDDP) against oral squamous cell carcinoma *in vitro*

Omar M. Alaufi[1, 2], Abdulwahab Noorwali[1], Fatheya Zahran[3], Ahmed M. Al-Abd[*4, 5, 6], Safia Al-Attas[7]

[1]Departement of Clinical Biochemistry, Faculty of Medicine, King Abdulaziz University, Jeddah, Saudi Arabia
[2]General Directorate of Medical Services, Ministry of Interior, Riyadh, Saudi Arabia
[3]Oral medicine and Periodontology Department, Faculty of Dentistry, Cairo University, Cairo , Egypt
[4]Pharmacology Department, Medical Division, National Research Centre, Cairo, Egypt
[5]Departement of Pharmacology and Toxicology, Faculty of Pharmacy, King Abdulaziz University, Jeddah, Saudi Arabia
[6]Nawah Scientific, Mokkatam, Cairo, Egypt
[7]Department of Oral Diagnostic Sciences, Faculty of Dentistry, King Abdulaziz University, Jeddah, Saudi Arabia

[*]Corresponding authors:
Ahmed M. Al-Abd, Pharmacology and Toxicology Dept. Faculty of Pharmacy, King Abdulaziz University, Jeddah 21589, P.O. 80384
Tel: +966-(0)2-640-0000/Ext 21125
Fax: +966-(0)2-695-1696
E-mail: amalabd@kau.edu.sa

Raw data Alaufi et al., 2017

UMSCC-14 cell line

Bcl2

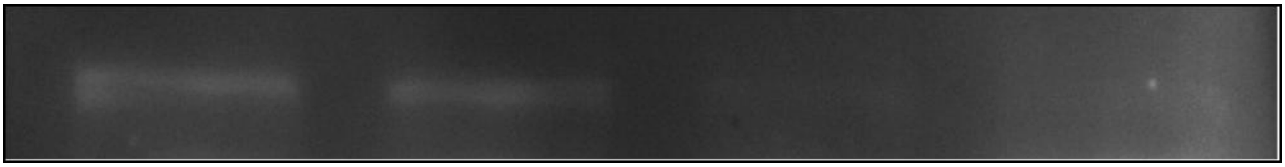

Caspase-9

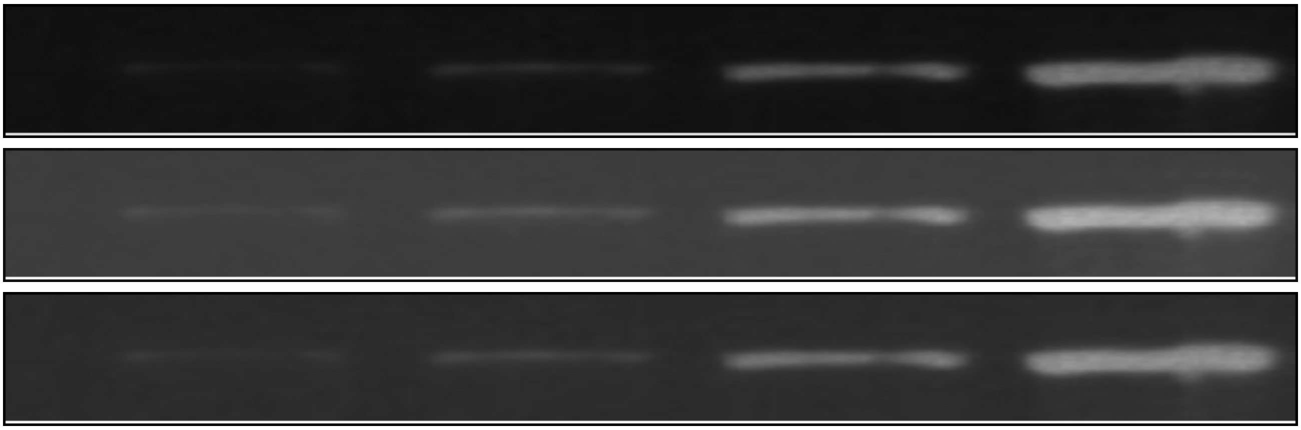

P53

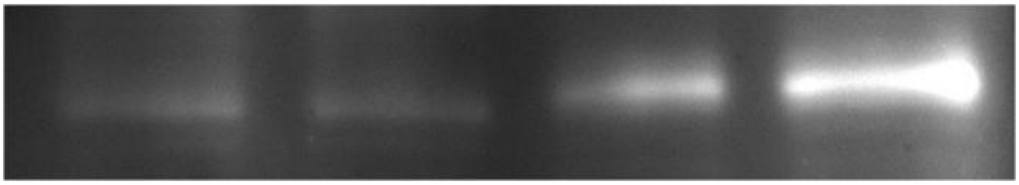

GAPDH

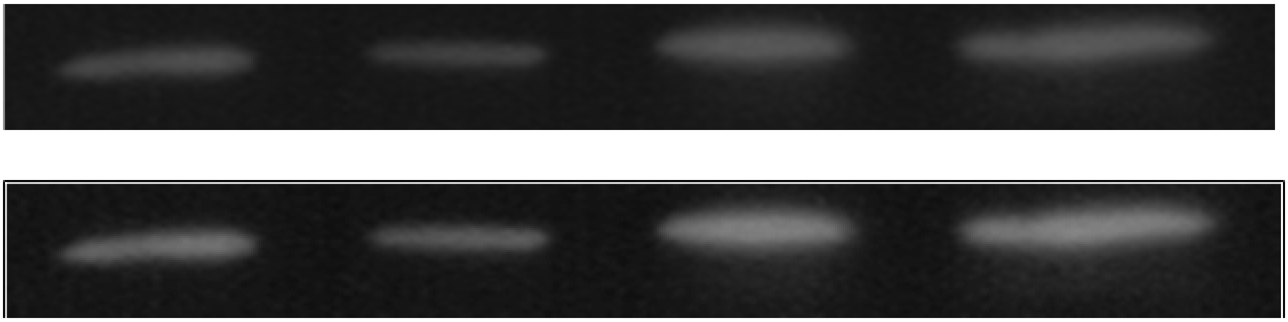

OEC cell line

Bcl2

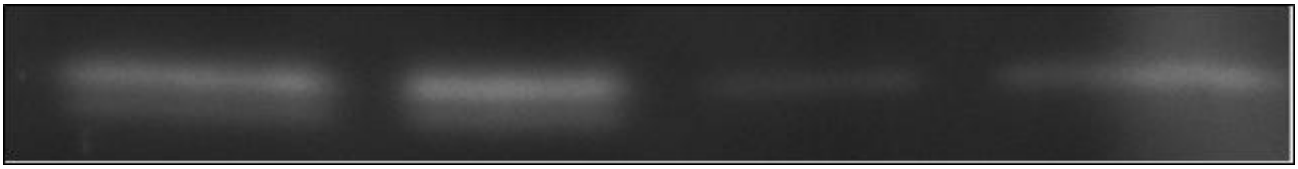

Caspase-9

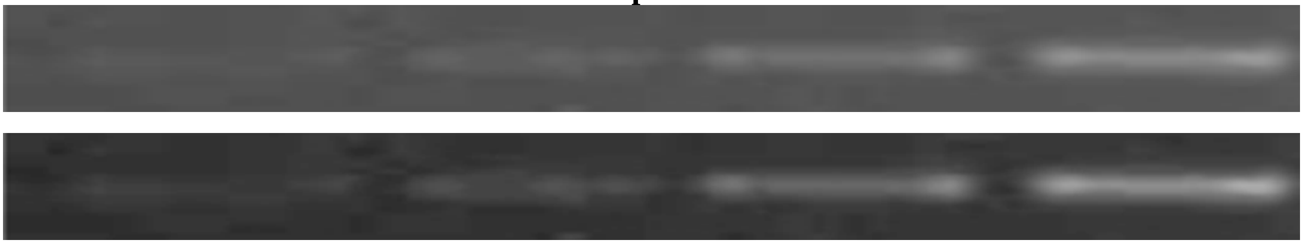

P53

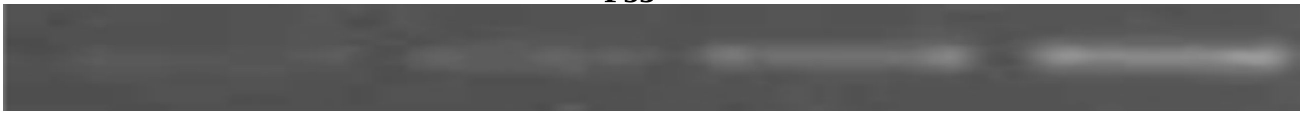

GAPDH

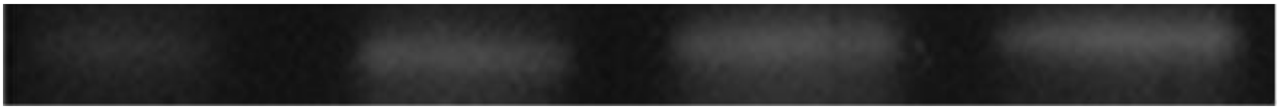

Supplement: Supplementary file 1 — Supplementary File [file 41598_2017_13357_MOESM1_ESM.pdf]
